# Association of Polymorphisms of Inflammatory-Relevant Genes with Cancer Risk

**DOI:** 10.3390/cimb48060548

**Published:** 2026-05-23

**Authors:** Sara AlSrayea, Maryam H. Alrashid, Nasmah K. Bastaki, Jasem Al-Barrak

**Affiliations:** 1 Department of Biological Sciences, College of Science, Kuwait University, Sabah Al Salem University City, P.O. Box 5969, Safat 13060, Shadadiya, Kuwait; s.alsrayea@ku.edu.kw (S.A.); nasmah.bastaki@ku.edu.kw (N.K.B.); 2 Kuwait Cancer Control Center, Kuwait City 70030, Kuwait; jaalbarrak@moh.gov.kw

**Keywords:** colorectal cancer, non-Hodgkin lymphoma, inflammatory genes, NF-kB, TNF-α, SNPs, screening, biomarkers

## Abstract

Colorectal cancer (CRC) and non-Hodgkin lymphoma (NHL) are among the most prevalent cancer types globally by incidence and mortality. Both types are influenced differentially by chronic inflammation. Central to this inflammation are inflammatory genes that are meticulously regulated by nuclear factor kappa B (NF-κB) and tumor necrosis factor-α (TNF-α). NF-κB is negatively regulated by IκBα (encoded by *NFKBIA*), while TNF-α’s actions can be modulated by ghrelin (encoded by *GHRL*). We investigated four single nucleotide polymorphisms (SNPs) in *NFKB1* (rs4648068), *NFKBIA* (rs2233406), *TNF-α* (rs1800629), and *GHRL* (rs1629816) as biomarkers for CRC and NHL risk in a cohort of Kuwaiti individuals. DNA samples from patients and controls were collected and genotyped for all SNPs, and their association with CRC or NHL risk was assessed. While rs4648068 showed a modest association with increased CRC risk, it had no significant impact on NHL risk. Conversely, rs2233406 increased NHL risk without affecting CRC risk. Interestingly, while rs1800629 showed a protective effect against NHL, it showed an increased risk for CRC. Finally, rs1629816 was associated with greater NHL but not CRC risk. Our findings suggests that variations of these inflammatory genes may be useful indicators for predicting cancer risk but might have unpredictable effects on cancer susceptibility, depending on the cancer type.

## 1. Introduction

Cancer continues to be a major global public health challenge. Colorectal cancer (CRC) and non-Hodgkin lymphoma (NHL) are major public health concerns, ranking among the top ten most common cancers worldwide [1]. Both CRC and NHL are influenced by inflammation, albeit through different mechanisms [2,3]. Chronic inflammation is a key contributor to cancer development and progression by promoting cytokine release, oxidative stress, angiogenesis, immune suppression, and activation of pathways such as Nuclear factor kappa B (NF-κB) and Signal Transducer and Activator of Transcription 3 (STAT3) [4,5]. Persistent inflammatory conditions create a tumor-promoting environment through mechanisms such as DNA damage, immune system modifications, and increased angiogenesis [6]. In epithelial cancers such as colorectal cancer, persistent inflammation can damage epithelial barriers, induce DNA damage, and support tumor growth, invasion, and metastasis through cytokines including tumor necrosis factor-alpha (TNF-α), interleukin-6 (IL-6), and interleukin-1β (IL-1β) [7,8]. In NHL, inflammation is more closely linked to chronic immune activation and dysregulation of the lymphoid microenvironment, where cytokines produced by malignant and stromal immune cells support lymphoma-cell survival, migration, and immune escape [9,10]. Thus, inflammation contributes to both CRC and NHL, but through distinct tissue-specific mechanisms. NF-κB is a transcription factor that plays a critical role in inflammatory responses and acts as a promoter of tumor development and progression by inducing the expression of various pro-inflammatory genes as well as regulating other cellular processes such as immune response, proliferation, and cell survival [11,12]. Moreover, NF-κB is activated by various pathways, one of which is the TNF-α pathway. TNF-α is a pro-inflammatory cytokine that plays an essential role in regulating immune responses and inflammation. It is produced in response to various stimuli such as infections, autoimmune processes, or tissue injury [13]. Interestingly, TNF-α has a paradoxical role in cancer since it can act as both a tumor suppressor and a tumor promoter based on the expressed TNF-α receptor (TNFR), where higher TNFR1 is mostly associated with tumor-suppressor effect while higher TNFR2 induces more of a tumor-promoter effect [14]. This “switch” is highly cell-type-dependent, where in epithelial (tumor) cells TNF-α can promote NF-κB-linked epithelial–mesenchymal transition (EMT) and invasion under chronic inflammatory conditions, whereas in tumor endothelial cells it can act as a tumor suppressor by promoting apoptosis [15,16]. Nevertheless, elevated TNF-α levels have been detected in several malignancies, including breast, lung, CRC, and hematologic cancers, where it correlated with poor prognosis and therapy resistance [17].

TNF-α is inhibited by ghrelin, a 28-amino-acid peptide hormone encoded by *GHRL* and released by the stomach cells. Research indicates that ghrelin can downregulate pro-inflammatory cytokines, including TNF-α [18,19]. Recent studies show that ghrelin might have a potential role in cancer development and progression since it can stimulate cell proliferation and metastasis [20]. However, ghrelin’s actions in cancer appear to be highly context-dependent, with reported effects varying by tumor type, which may explain why both pro- and anti-tumor effects are described [21].

Single nucleotide polymorphisms (SNPs) are the most common form of genetic variation in the human genome [22]. Identifying specific SNPs could help recognize high-risk individuals, allowing for early intervention and personalized monitoring or treatment [23]. Studies show that genetic predispositions are involved in CRC and NHL development [24,25]. SNPs in the NF-κB subunit 1 gene (*NFKB1*), *TNF-α* and *GHRL* genes have been linked to changes in NHL and CRC risk. The *NFKB1* rs4648068 is an A > G SNP located in intron 12 of the *NFKB1* gene, which encode the NF-κB p105 precursor that is processed into the p50 subunit. This SNP has been linked to cancer susceptibility in several populations, particularly to cancers where NF-κB signaling is implicated in inflammation and tumorigenesis [26,27,28]. Previous functional work showed that the GG genotype increased *NFKB1* transcriptional activity and NF-κB1/p50 expression, although more recent studies mainly support its inflammatory disease association rather than providing new mechanistic validation [28,29]. Additionally, rs2233406, a C > T SNP in the promoter region of the *NFKBIA* gene, has been investigated in various cancers, often with inflammation-related etiologies [30,31]. *NFKBIA* encodes IκBα, an inhibitory protein that binds NF-κB and keeps it inactive until IκBα is phosphorylated and degraded, releasing NF-κB to enter the nucleus and induce *NFKBIA* gene expression as negative feedback [32]. Available functional evidence suggests that promoter variants linked to this locus may reduce *NFKBIA*/IκBα expression and thereby enhance NF-κB-mediated inflammatory responses [33].

The *TNF-α* rs1800629 is an SNP in the promoter region. Functional and clinical data suggest that the A allele can increase *TNF-α* transcriptional activity and is often associated with higher TNF-α levels compared with the G allele [34]. In addition, rs1800629 has been widely studied as a pharmacogenetic (drug-response) marker for anti-inflammatory TNF-α blocker drugs, with several cohorts/reviews reporting more non-responders among A-allele carriers. However, results were inconsistent across diseases and drugs, since some studies/meta-analyses found weak or no association, especially when stratified by specific TNF-blocker [35,36]. This SNP has shown varying effects on cancer risk; The International Lymphoma Epidemiology Consortium study found this variant to be significantly associated with the risk for NHL, while other studies found that this association might increase, decrease or not be observed, depending on the population studied [37]. On the other hand, the relationship with CRC risk is less clear: multiple meta-analyses generally report no consistent association with overall CRC susceptibility, but they do note heterogeneity across populations [38,39]. In a study by Li et al. 2017, rs1800629 was not associated with overall CRC risk, but it showed associations with larger tumor size and distant metastasis, especially in rectal cancer [40].

Although several recent studies showed that ghrelin may play in carcinogenesis, however, studies failed to show a consistent association of its genetic variants with the risk for CRC or NHL. Nevertheless, a synonymous SNP in the promotor region of the *GHRL* gene, rs1629816, has been reported to be associated with an increased risk for certain cancers, including NHL breast cancer, although no functional effect of the SNP has been established [41,42,43].

Inflammation-related genetic associations may differ across populations and across tumor types, and the extent to which candidate variants in *NFKB1*, *NFKBIA*, *TNF-α*, and *GHRL* show shared or cancer-type-specific associations in Kuwaitis remains unclear. Understanding the SNPs’ association with specific cancer risk will steer us to determine individuals with a high risk of CRC and NHL, thus tailoring strategies for better cancer screening and prevention. Our research examined the selected mentioned SNPs in patients with CRC and others with NHL in a group of Kuwaitis and compared the genotype and allele distribution between those two groups and a control group. This is to establish the presence or absence of an association between these SNPs with the risk for CRC and NHL. We analyzed CRC and NHL in parallel since both malignancies involve inflammatory signaling, but they arise in biologically distinct tissue contexts that may modify the effect of the same variant. The aim of this case–control study was therefore to compare the genotype and allele distributions of four candidate SNPs in Kuwaiti patients with CRC, NHL, and cancer-free controls, and to estimate their associations with disease risk. This will enable us to identify whether any of the variants can be considered a risk-associated variant, which upon further validation in future studies can be established as a biomarker for risk screening of the disease.

## 2. Materials and Methods

### 2.1. Study Population

A total of 559 genomic DNA samples from Kuwaiti volunteers aged 18–75 years of both sexes were analyzed in this study. The study cohort consisted of three groups: CRC patients, NHL patients, and control individuals. The NHL (141) and CRC (144) patients were diagnosed and recruited at the Kuwait Cancer Care Center (KCCC). Inclusion criteria included Kuwaiti individuals aged 18 years or older who provided informed consent and a blood sample. For the patient groups, participants were required to have a confirmed diagnosis of CRC or NHL, while controls were required to have no history of cancer diagnosis. Individuals were excluded if they were non-Kuwaiti, younger than 18 years old, lacked a confirmed diagnosis for the patient groups, or did not provide informed consent or a blood sample at the time of interview. Controls were cancer-free volunteers recruited from general hospitals and polyclinics in Kuwait City Governorate during routine check-up visits. They were selected to match the patient groups as closely as possible by age and sex and had no previous cancer diagnosis. All samples have been collected from multiple hospitals across Kuwait, with clinical and demographic data recorded in a dedicated database. Each participant provided written informed consent for use of their blood samples and data in research. Ethical approval for this study was obtained from the Kuwait University College of Science Bioethics Committee and the Ministry of Health (Kuwait Institute for Medical Specialization), in accordance with the principles of the Declaration of Helsinki.

### 2.2. Demographic and Clinical Characteristics of Study Participant

The study included 274 control participants, 144 CRC patients, and 141 NHL patients (Table 1). Sex distribution was comparable across all groups, with males and females nearly equally represented, and no significant differences were observed (*p* = 0.759). The mean age of CRC patients (59.77 ± 12.21 years) was higher than that of controls (50.38 ± 13.46 years) and NHL patients (53.57 ± 15.52 years). BMI was slightly lower in CRC (27.5 ± 7.72) and NHL patients (27.83 ± 7.50) compared to controls (29.03 ± 6.7).

### 2.3. DNA Extraction and Quantification

Blood samples were collected in EDTA-coated tubes (Advance Medical Co.,Ltd., Riyadh, Saudi Arabia) and genomic DNA was extracted from peripheral blood leukocytes for all samples using the Gentra^®^ Puregene^®^ Blood DNA Extraction kit (Qiagen, Hilden, Germany) according to the manufacturer’s instructions. All DNA samples were assessed for concentration and purity using a NanoDrop™ Eight UV-Vis spectrophotometer (Thermo Fisher Scientific, Waltham, MA, USA).

### 2.4. SNP Selection and Genotyping by Real Time PCR

Four candidate SNPs in inflammatory genes, *NFKB1* (C__11345289_20), *NFKBIA* (C_____73867_10), *TNF-α* (C___7514879_10), and *GHRL* (C___3114629_10), were chosen for genotypic analysis, as shown in (Table 2). Genotyping was performed using predesigned TaqMan^®^ SNP Genotyping Assays (Applied Biosystems, Thermo Fisher Scientific, Waltham, MA, USA) on QuantStudio™ Real-Time PCR System (Applied Biosystems, Thermo Fisher Scientific, Waltham, MA, USA) according to the manufacturer’s instructions. Genotype calls were assigned using TaqMan allelic discrimination plots generated by the QuantStudio system, and the genotype clusters were reviewed manually to confirm appropriate separation of homozygous, heterozygous, and no-template control groups. For quality control, all tests were performed with multiple negative controls and random samples were analyzed in duplicates to ensure the accuracy of the results.

### 2.5. Statistical Analysis

Hardy–Weinberg equilibrium (HWE) was assessed using a web-based Pearson’s chi-square test calculator, with *p* < 0.05 indicating deviation from equilibrium within a given group. Statistical analyses were performed using IBM SPSS Statistics (version 25; IBM Corp., Chicago, IL, USA). To evaluate associations between each SNP and disease susceptibility, genotype distributions were first compared between cases and controls (CRC vs. controls and NHL vs. controls) using Pearson’s chi-square test. To estimate the association between each SNP and disease risk while accounting for potential confounding, we performed multivariate and logistic regression analysis to estimate odds ratios (ORs) for genotype effects under codominant (two indicator variables for heterozygous and minor-homozygous genotypes), dominant (carrier versus non-carrier of the minor allele), and recessive (minor-homozygous genotype versus all others) genetic models. Multivariable models included age, sex and BMI as covariates. Results were reported as ORs with 95% confidence intervals (CIs), and statistical significance was assessed as *p* < 0.05, adjusted to *p* < 0.0125 after Bonferroni correction.

## 3. Results

### 3.1. Genotypic and Allelic Frequencies

All study participants were genotyped for the four selected SNPs. Minor allele frequency (MAF) was determined for the SNPs, and all the genetic variants had MAF > 0.05 (Table 2). The genotype and allele frequencies are summarized in (Table 3). For all the SNPs, the major allele was the most frequent allele in both controls and patients, except the NHL samples in rs1800629 and rs1629816. A significant difference was observed in the genotype frequency between the controls and patients’ group for rs4648068 in CRC (*p* = 0.01), rs2233406 in NHL (*p* = 0.012, rs1800629 in CRC (*p* ≤ 0.001) and in NHL (*p* ≤ 0.001), and rs1629816 in NHL (*p* ≤ 0.001). These unadjusted associations highlight potential associations between specific variants and disease susceptibility, especially for rs1800629. HWE was assessed for each SNP within the control, CRC, and NHL groups.

### 3.2. Analysis of SNP Effects on CRC and NHL Risk

Associations between the selected SNPs and the risks of CRC and NHL were estimated using logistic regression models adjusted for age, sex and BMI (Table 4 and Table 5, respectively). For rs4648068, in the codominant model, individuals with the AG genotype had a significantly increased risk of CRC (*p* = 0.032, OR, 1.65; 95% CI, 1.04–2.60); however, the other models showed no association (Table 4). For rs2233406, the GA genotype was significantly associated with increased NHL risk in the codominant model (*p* = 0.009, OR, 1.79; 95% CI, 1.16–2.76) but not the dominant and recessive models (Table 5). Moreover, rs1800629 showed an association with an increased risk of CRC in all genetic models (Table 4). The GA and AA were associated with an increased risk in the codominant model (*p* < 0.001, OR, 9.42; 95% CI, 3.84–23.12 and *p* < 0.001, OR, 15.47; 95% CI, 6.77–35.36, respectively). In the dominant model, GA + AA vs. GG was associated with an increased risk of CRC (*p* < 0.001, OR, 13.37; 95% CI, 5.94–30.10), while the recessive model showed that AA vs. GA + GG yielded an OR of 4.1; 95% CI, 2.58–6.50, *p* < 0.001, indicating strong additive and recessive effects.

In NHL, rs1800629 showed an inverse association where the AA genotype conferred a markedly reduced risk (*p* < 0.001, OR, 0.1; 95% CI, 0.05–0.22), and the dominant model (GA + AA vs. GG) yielded an OR of 0.3; 95% CI, 0.20–0.47, *p* < 0.001, indicating a protective effect of the A allele. Also, rs1629816 was associated with NHL risk as the AA homozygotes genotype had a significantly higher risk (*p* < 0.001, OR, 3.47; 95% CI, 1.85–6.49) in the codominant model, and both dominant (GA + AA vs. GG) and recessive (AA vs. GA + GG) models were significant (*p* < 0.001, OR, 2.79; 95% CI, 1.59–4.91 and *p* = 0.011, OR, 2.02; 95% CI, 1.17–3.50, respectively) (Table 5).

## 4. Discussion

In this study, we examined four SNPs in inflammatory-relevant genes—rs4648068 in *NFKB1*, rs2233406 in *NFKBIA*, rs1800629 in *TNF-α* and rs1629816 in ghrelin—for their association with CRC and NHL susceptibility in a cohort of Kuwaiti individuals. The main pattern emerging from the dataset was that not all variants behaved consistently across the two cancers, suggesting possible cancer-type-specific effects within inflammatory pathways. Our most notable novel finding is that the *TNF-α* promoter SNP rs1800629 showed opposite associations with CRC and NHL in our cohort, emerging as a particularly significant marker (Table 4 and Table 5). While the A allele was associated with an increased CRC risk (*p* < 0.001, OR, 9.42; 95% CI, 3.84–23.12 for GA and *p* < 0.001, OR, 15.47; 95% CI, 6.77–35.36 for AA), interestingly it was associated with a decreased risk for NHL (*p* < 0.001, OR, 0.1; 95% CI, 0.05–0.22).

Biologically, the positive association with CRC risk is related to TNF-α’s role as a pro-inflammatory cytokine that can drive tumor-promoting inflammation in the colon [44]. Chronic colonic inflammation is a known risk factor for CRC, and TNF-α is one of the key mediators of inflammation-induced carcinogenesis in the gut [6]. In a large case–control study in China, no association between the overall CRC risk and *TNF-α* rs1800629/G variant was found; however, that study did observe that those carrying the A allele had significantly worse disease characteristics including larger tumor size and a higher risk for distant metastasis [40]. Our observation of a strong association (*p* < 0.001, OR, 13.37; 95% CI, 5.94–30.10 under a dominant model) with CRC risk in Kuwait is significant and suggests that *TNF-α* rs1800629/A variant merits further investigation as a potential genetic risk factor for CRC in other regional populations. In contrast, the *TNF-α* rs1800629/A variant was associated with a lower risk of NHL in our study, which is consistent with what has previously been reported [45]. TNF-α can act as a tumor suppressor under certain conditions by promoting immune responses and apoptotic death of cancer cells [46]. However, the research on *TNF-α* polymorphisms in lymphoma risk does not clearly document a protective effect of the A allele. A meta-analysis by Zhai et al. (2014) noted an increase in NHL risk for carriers of the *TNF-α* rs1800629/A variant in Caucasians but decreased risk of NHL in Asians, although results have varied [47]. These contrasting associations may reflect epistatic interactions and context-dependent regulation of TNF-α, whereby the effect of the *TNF-α* rs1800629/A variant is modified by other immune-related loci and tissue-specific inflammatory environments rather than acting independently. Such gene–gene interactions and regulatory variability can lead to non-random genotype distributions in the general population, resulting in deviation from HWE in controls without implying genotyping error [48,49]. Similar deviations have been reported in inflammatory and cancer-related SNPs and are thought to arise from selection pressures or pleiotropic effects, particularly in immune pathway genes such as *TNF-α* [50,51].

The rs1629816, an SNP in the promoter region of *GHRL*, showed no effect on CRC risk but was linked to higher NHL risk (AA genotype *p* < 0.001, OR, 3.47; 95% CI, 1.85–6.49). The A allele was more common in NHL patients than controls (AA frequency = 0.13% in cases vs. 0.08% in controls, dominant model *p* < 0.001, OR, 2.79; 95% CI, 1.59–4.91), suggesting that this variant contributes to higher NHL susceptibility. Notably, this SNP has been previously implicated in lymphoma risk [52]. A meta-analysis in ghrelin polymorphisms found that certain *GHRL* polymorphisms influenced cancer susceptibility in Caucasian populations. In that analysis, two *GHRL* gene SNPs (rs696217 and rs2075356) showed significant protective associations, whereas other *GHRL* SNPs (rs4684677 and rs572169) were linked to increased risk of breast cancer [43]. These findings highlight that those alterations in the *GHRL* gene are associated with cancer risk in an inverse effect depending on the variant and cancer type.

The intronic *NFKB1* rs4648068 showed a modest association with elevated CRC risk (AG genotype *p* = 0.032, OR, 1.65; 95% CI, 1.04–2.60) before Bonferroni correction; however, after the correction the significance was lost. No significant impact of this SNP was found on NHL, whereas the *NFKBIA* rs2233406 was associated with increased NHL risk (*p* = 0.009, OR, 1.79; 95% CI, 1.16–2.76) for the GA genotype but not with CRC. These results suggest that pro-inflammatory genetic variants may promote or suppress tumor development depending on the tissue context, highlighting the interplay between chronic inflammation and carcinogenesis. NF-κB is a master regulator of inflammation, cell survival, and proliferation, and its chronic activation is known to create a tumor-promoting environment [6]. The *NFKB1* rs4648068 has been shown to enhance NFKB1 expression and activity in previous studies. Chen et al. (2015) demonstrated that gastric cancer patients with the rs4648068 GG genotype had higher NF-κB levels and more aggressive tumor cell behavior, linking this SNP to increased proliferation and motility of cancer cells [53]. Consistent with that mechanism, our data indicate that carrying the G allele modestly elevates CRC risk. This also aligns with a previous study in which the G allele was associated with an increased risk of ovarian cancer [28]. Nonetheless, the absence of association with NHL in our study is consistent with a meta-analysis that found no overall cancer susceptibility effect of rs4648068 when pooling multiple cancer types and indicates that any contribution of this variant to lymphoma risk is likely small [54]. The *NFKBIA* rs2233406 variant showed no association with CRC and a weak association with NHL risk in our data. A prior meta-analysis on *NFKBIA* polymorphisms has concluded rs2233406 was not a significant risk factor for overall cancer susceptibility [55]. Further studies with larger CRC and NHL subgroups are needed to confirm if this promoter SNP influences cancer risk.

In our control group, we detected a deviation from HWE for the investigated SNPs. Similar observations have been reported in published association studies of inflammatory-related polymorphisms, where control samples fell out of HWE yet the findings remained valid [50,56]. Deviation from HWE alone does not invalidate our association results. HWE assumes an idealized population (random mating, no selection, no substructure), conditions which are often violated in reality [57,58]. Selective forces or pleiotropy effects acting on cytokine and immune-regulatory genes can influence genotype distribution in the general population, particularly for variants involved in inflammatory and immune responses [48]. As a result, immune-related variants under balancing selection or linked to unrecognized traits may deviate from HWE in control samples even with accurate genotyping [49]. Researchers have emphasized that HWE deviations can reflect population-genetic factor (e.g., natural selection) rather than technical error [57]. In summary, the observed HWE deviation in our Kuwaiti control group is possibly due to biological and population-genetic factors and does not compromise the validity of our SNP association reported in this study. However, these findings should be interpreted cautiously, and as a result, we treat HWE deviation as a limitation of the present dataset.

We acknowledge several limitations in our study. The sample size, especially for subgroup analyses, was modest, with 144 CRC cases and 141 NHL cases, which limited our statistical power. Since it is a retrospective study, this may introduce selection bias and missing data. In addition, incomplete information on key risk factors (e.g., diet, smoking, infection history) of the study cohort may still influence the results.

## 5. Conclusions

The highlight of our study is the finding that the risk-associated SNPs rs1800629 in *TNF-α* and rs1629816 in *GHRL* appear to exert opposing effects on CRC and NHL susceptibility, highlighting their potential roles in cancer-type-specific immune or inflammatory pathways. If validated in further studies and more populations, these SNPs could serve as predictive genetic biomarkers for cancer risk. Larger multicenter studies with rigorous genotyping quality control and functional follow-up are needed before any clinical application can be considered.

## Figures and Tables

**Table 1:** Demographic and clinical characteristics of study participants.

Characteristics	Control Cases (*n* = 274)	CRC Cases (*n* = 144)	NHL Cases (*n* = 141)
**Sex**			
Male	128 (46.7%)	65 (45.1%)	66 (46.8%)
Female	146 (53.3%)	79 (54.9%)	75 (53.2%)
**Age** (Mean ± Std)	50.38 ± 13.46	59.77 ± 12.21	53.57 ± 15.52
**BMI** (Mean ± Std)	29.03 ± 6.7	27.5 ± 7.72	27.83 ± 7.50

Values provided are the mean ± standard deviation or number of patients (*n*) (%). Body mass index (BMI); colorectal cancer (CRC); non-Hodgkin lymphoma (NHL).

**Table 2:** Variants summary of the selected genes.

Gene Name	SNP	Alleles **	Gene Location	Chromosomal Localization	MAF *
*NFKB1*	rs4648068	A > G	Intron11 region	Chr.4: 102597148 on GRCh38	0.311
*NFKBIA*	rs2233406	G > A	Intron region	Chr.14: 35405593 on GRCh38	0.262
*TNF-α*	rs1800629	G > A	Promoter region	Chr.6: 31575254 on GRCh38	0.128
*GHRL*	rs1629816	G > A	Promoter region	Chr.3: 10294607 on GRCh38	0.42

* MAF = Minor Allele Frequency (Extracted from Ensembl). ** Reference allele > variant allele.

**Table 3:** Genotype distributions and allele frequencies of the investigated SNPs across the study cohort.

SNP	Control *n* = 274 (%)	CRC Patients *n* = 144 (%)	*p*	NHL Patients *n* = 141 (%)	*p*
*NFKB1*					
**rs4648068**					
AA	146 (53.3%)	63 (43.8%)		63 (44.7%)	
AG	89 (32.5%)	68 (47.2%)	**0.01** *	60 (42.6%)	0.126
GG	39 (14.2%)	13 (9.0%)		18 (12.8%)	
Allele **A** (%)	69.5%	67.4%		66%	
Allele **G** (%)	30.5%	32.6%		34%	
HWE	<0.001	0.375		0.534	
*NFKBIA*					
**rs2233406**					
GG	28 (10.2%)	10 (6.9%)		9 (6.4%)	
GA	83 (30.3%)	54 (37.5%)	0.239	63 (44.7%)	**0.012** *
AA	163 (59.5%)	80 (55.6%)		69 (48.9%)	
Allele **A** (%)	74.6%	74.3%		71.3%	
Allele **G** (%)	25.4%	25.7%		28.7%	
HWE	<0.001	0.83		0.279	
*TNF-α*					
**rs1800629**					
GG	119 (43.4%)	7 (4.9%)		100 (70.9%)	
GA	57 (20.8%)	32 (22.2%)	<**0.001** *	32 (22.7%)	<**0.001** *
AA	98 (35.8%)	105 (72.9%)		9 (6.4%)	
Allele **A** (%)	46.2%	84%		17.7%	
Allele **G** (%)	53.8%	16%		82.3%	
HWE	<0.001	0.039		0.008	
*GHRL*					
**rs1629816**					
GG	76 (27.7%)	36 (25.0%)		56 (39.7%)	
GA	120 (43.8%)	61 (42.4%)	0.651	67 (47.5%)	<**0.001** *
AA	78 (28.5%)	47 (32.6%)		18 (12.8%)	
Allele **A** (%)	50.4%	53.8%		36.5%	
Allele **G** (%)	49.6%	46.2%		63.5%	
HWE	0.04	0.076		0.769	

Values given are the number of individuals (***n***) (%). * *p* < 0.05 (highlighted in bold) indicates evidence of association. In HWE, *p*-value < 0.05 indicates a significant deviation from HWE.

**Table 4:** Association between selected SNPs and CRC risk.

SNP	OR (CI 95%)	*p*-Value
**rs4648068**		
**Codominant**		
AA	1	
AG	1.65 (1.04–2.60)	**0.032**
GG	0.78 (0.37–1.61)	0.498
**Dominant**		
AA	1	
AG + GG	1.39 (0.91–2.14)	0.13
**Recessive**		
AA + AG	1	
GG	0.62 (0.31–1.25)	0.181
**rs2233406**		
**Codominant**		
GG	1	
GA	1.29 (0.81–2.05)	0.277
AA	0.88 (0.39–1.97)	0.758
**Dominant**		
GG	1	
GA + AA	1.2 (0.78–1.84)	0.415
**Recessive**		
GG + GA	1	
AA	0.8 (0.37–1.76)	0.582
**rs1800629**		
**Codominant**		
GG	1	
GA	9.42 (3.84–23.12)	**<0.001**
AA	15.47 (6.77–35.36)	**<0.001**
**Dominant**		
GG	1	
GA + AA	13.37 (5.94–30.10)	**<0.001**
**Recessive**		
GG + GA	1	
AA	4.1 (2.58–6.50)	**<0.001**
**rs1629816**		
**Codominant**		
GG	1	
GA	0.79 (0.48–1.31)	0.366
AA	0.9 (0.51–1.60)	0.729
**Dominant**		
GG	1	
GA + AA	0.85 (0.54–1.34)	0.478
**Recessive**		
GG + GA	1	
AA	1.08 (0.63–1.85)	0.78

The analysis was adjusted for age, sex, and BMI. Statistically significant associations (*p* < 0.05) are highlighted in bold.

**Table 5:** Associations between selected SNPs and NHL risk.

SNP	OR (CI 95%)	*p*-Value
**rs4648068**		
**Codominant**		
AA	1	
AG	1.53 (0.98–2.38)	0.061
GG	1.06 (0.56–1.99)	0.866
**Dominant**		
AA	1	
AG + GG	1.39 (0.92–2.09)	0.12
**Recessive**		
AA + AG	1	
GG	0.88 (0.48–1.61)	0.673
**rs2233406**		
**Codominant**		
GG	1	
GA	1.79 (1.16–2.76)	**0.009**
AA	1.01 (0.45–2.26)	0.985
**Dominant**		
GG	1	
GA + AA	1.46 (0.97–2.21)	0.071
**Recessive**		
GG + GA	1	
AA	0.99 (0.45–2.20)	0.979
**rs1800629**		
**Codominant**		
GG	1	
GA	0.65 (0.39–1.09)	0.101
AA	0.1 (0.05–0.22)	**<0.001**
**Dominant**		
GG	1	
GA + AA	0.3 (0.20–0.47)	**<0.001**
**Recessive**		
GG + GA	1	
AA	0.12 (0.06–0.24)	**<0.001**
**rs1629816**		
**Codominant**		
GG	1	
GA	1.08 (0.63–1.83)	0.781
AA	3.47 (1.85–6.49)	**<0.001**
**Dominant**		
GG	1	
GA + AA	2.79 (1.59–4.91)	**<0.001**
**Recessive**		
GG + GA	1	
AA	2.02 (1.17–3.50)	**0.011**

The analysis was adjusted for age, sex, and BMI. Statistically significant associations (*p* < 0.05) are highlighted in bold.

## Data Availability

All data generated or analyzed during this study are available upon request from the corresponding author.
